# Challenges in sodium intake reduction and meal consumption patterns among participants with metabolic syndrome in a dietary trial

**DOI:** 10.1186/1475-2891-12-163

**Published:** 2013-12-18

**Authors:** Jinsong Wang, Barbara C Olendzki, Nicole M Wedick, Gioia M Persuitte, Annie L Culver, Wenjun Li, Philip A Merriam, James Carmody, Hua Fang, Zhiying Zhang, Gin-Fei Olendzki, Liang Zheng, Yunsheng Ma

**Affiliations:** 1 Department of Preventive Medicine, Medical School of Yangzhou University, Yangzhou, Jiangsu, China; 2 Division of Preventive and Behavioral Medicine, Department of Medicine, University of Massachusetts Medical School, Worcester, MA, USA; 3 Division of Biostatistics and Health Services Research, Department of Quantitative Health Science, Clinical and Population Health Research Doctoral Program, University of Massachusetts Medical School, Worcester, MA, USA; 4 Division of Biostatistics and Health Services Research, Department of Quantitative Health Science, University of Massachusetts Medical School, Worcester, MA, USA; 5 Tongji University Medical School, Shanghai, China

**Keywords:** Sodium, Diet, Metabolic syndrome, Meal type, Meal location

## Abstract

**Background:**

Dietary guidelines suggest limiting daily sodium intake to <2,300 mg for the general population, and <1,500 mg/d for those with certain cardiovascular risk factors. Despite these recommendations, few Americans are able to achieve this goal. Identifying challenges in meeting these guidelines is integral for successful compliance. This analysis examined patterns and amount of daily sodium intake among participants with metabolic syndrome enrolled in a one-year dietary intervention study.

**Methods:**

Two hundred forty participants with metabolic syndrome enrolled in a dietary intervention trial to lose weight and improve dietary quality. Three 24-hour dietary recalls were collected at each visit which provided meal patterns and nutrient data, including sodium intake. A secondary data analysis was conducted to examine sodium consumption patterns at baseline and at one-year study visits. Sodium consumption patterns over time were examined using linear mixed models.

**Results:**

The percentage of meals reported eaten in the home at both baseline and one-year follow-up was approximately 69%. Follow-up for the one-year dietary intervention revealed that the participants who consumed sodium greater than 2,300 mg/d declined from 75% (at baseline) to 59%, and those that consumed higher than 1,500 mg/d declined from 96% (at baseline) to 85%. Average sodium intake decreased from 2,994 mg at baseline to 2,558 mg at one-year (*P* < 0.001), and the sodium potassium ratio also decreased from 1.211 to 1.047 (*P* < 0.001). Sodium intake per meal varied significantly by meal type, location, and weekday, with higher intake at dinner, in restaurants, and on weekends. At-home lunch and dinner sodium intake decreased (*P* < 0.05), while dinner sodium intake at restaurant/fast food chains increased from baseline to one-year (*P* < 0.05).

**Conclusion:**

Sodium intake for the majority of participants exceeded the recommended dietary guidelines. Findings support actions that encourage low-sodium food preparation at home and encourage public health policies that decrease sodium in restaurants and prepared foods.

## Introduction

High sodium intake is associated with many chronic diseases such as stroke and cardiovascular disease (CVD) [1-6]. It has been estimated that reducing sodium intake to <2300 mg/d may eliminate 11 million hypertension cases, save 18 billion health care dollars, and ultimately gain 312,000 quality-adjusted life years in the United States (U.S.) [7].

Dietary Guidelines for Americans (DGA) 2010 suggest a reduction in daily sodium intake to less than 2300 mg and even further reduction to 1500 mg for those 51 years and older, African-American, or individuals with hypertension, diabetes, or chronic kidney disease [8]. Despite these recommendations, sodium intake remains high in the U.S. with an average daily consumption of 3,266 mg for individuals 2 years of age and older [9].

Metabolic syndrome (MetS), characterized by central obesity, hypertension, dyslipidemia, and insulin resistance, is strongly associated with risk for type 2 diabetes and CVD [10,11]. The prevalence of metabolic syndrome in the U.S. increased from 27.9% in 1999 to 34.1% in 2006 [11-13]. Many studies indicate that high sodium intake is associated with MetS risk [14-17]; however, relationships between sodium intake and meal consumption patterns among MetS patients remain unclear. Therefore, to evaluate characteristics associated with sodium intake and variations in sodium intake patterns (by different locations and days of the week) we used data from our dietary intervention trial at baseline and at the end of the one-year intervention. We hypothesized an improvement in sodium intake patterns over our one-year dietary intervention.

## Methods

### Study sample

Details of the “Can Do” study methodology have been described elsewhere [18]. Briefly, recruitment was conducted at the University of Massachusetts Medical School (UMMS), Worcester, MA in May 2009 and was completed in February 2013. Study recruitment fliers were posted at the UMMS, local public libraries and churches; announcements were uploaded onto UMMS intranet; and recruitment advertisements were posted in local newspapers and on Craigslist. Institutional Review Board at UMMS approved this study. Eligibility criteria included the following: 1) Met diagnostic criteria for the MetS [19]; in brief, waist circumference >102 cm for men and 88 cm for women, triglycerides levels ≥150 mg/dL, high-density lipoprotein cholesterol (HDL-C) levels <40 mg/dL for men and <50 mg/dL for women, hypertension with systolic blood pressure ≥130 mmHg and diastolic blood pressure ≥ 85 mmHg, and fasting glucose concentrations ≥ 110 mg/dL; 2) Interest in losing weight and with body mass index (BMI) 30-40 kg/m^2^; 3) 20–70 y; 4) Telephone in the home or easy access to one; 5) Able to respond informed consent; 6) with physician’s approval to participate; 7) non-smoker (given nicotine’s weight and HDL-C suppression effect, and smoking cessation’s weight gain effect); and 8) speak, read and write English fluently. Individuals were excluded if they: 1) Had clinically diagnosed diabetes, or a fasting blood glucose levels ≥ 126 mg/dl at screening; 2) Had an acute coronary event within the 6 months prior to study; 3) Were pregnant or lactating; 4) Had polycystic ovary syndrome; 5) Planned to move out of the area within the 12-month study period; 6) Had a diagnosis of a medical condition that would preclude adherence to study dietary recommendations (e.g., inflammatory bowel disease, active diverticulitis, renal disease); 7) Had elevated depression [Center for Epidemiologic Studies Depression score > = 21] or suicidal ideation; 8) Were following a low-carbohydrate, high-fat dietary regimen such as the Atkins Diet; 9) Were participating in any current weight loss program; 10) Had bariatric surgery or was currently using weight loss education; or, 11) Had been diagnosed with an eating disorder (bulimia nervosa or binge eating). For the total of 1777 screened subjects, 1537 were excluded due to various reasons, i.e. telephone screening ineligible, screening visit ineligible, refused screening and/or baseline, and baseline visit ineligible.

A total of 240 obese subjects were randomized into one of the two conditions: 1) a high fiber diet (HF) or; 2) the American Heart Association (AHA) diet (control). Subjects following the HF diet received instructions toward achieving the goal of taking 30 g or more fiber daily from various foods; while those in the control group followed the AHA 2006 guidelines [20]. Briefly, based on a 2000 calories daily level, people should achieve 50-55% of calories from carbohydrate, 15-20% from protein, and 30-35% calories from fat. Specifically, people should 1) balance food intake and physical activity for a healthy weight; 2) consume at least 4.5 cups of fruit and vegetables; 3) consume at least two 3.5 ounces fish especially oily fish a week; 4) consume at least 50% grain intake as high fiber, whole-grain foods; 5) reduce sodium intake to less than 1500 mg/d; 6) minimize added sugar intake in foods and beverages and minimize hydrogenated fats content; 7) at least 4 servings of nuts, legumes, and seeds per week; 8) limited to no more than 2 servings of processed meats per week; 9) reduce saturated fat less than 7% of total energy intake, trans-fat to less than 1% of energy, and cholesterol to less than 300 mg/d by choosing lean foods vegetable alternative; 10) choose fat-free or low-fat (1% fat) dairy products; 11) choose moderate to no alcohol intake.

### Dietary assessment

To assess subjects’ dietary intake, three 24-hour dietary recalls (two week days and one weekend) were completed by study dietitians using the Nutritional Data System for Research (NDS-R, 2010–2012, Minneapolis, MN) at both baseline and one-year visits [21]. All participants received a food portion visual booklet prior to receiving the assessment calls to facilitate portion size estimation. Meal types were identified by participants as: breakfast, lunch, dinner or snack. Meal locations (where food was consumed) were chosen from selected options in NDS-R and grouped as follows: “At home” indicates meals eaten at home; “Away from home” includes eating meals at work, school, day care, friend’s home, community meal program, party, reception, sporting event, and other; and “Restaurant/fast food” includes eating meals at a restaurant, cafeteria, fast food chains, deli, take-out, or store. The 24-hour dietary recall provided variation of sodium intake by meal type, location, and day of the week. Sodium density was defined as sodium consumption per kilocalorie (kcal) of intake. Subjects were invited to complete a dietary attitudes questionnaire at both baseline and at one-year visit to query the importance of eating foods low in sodium.

### Sample size consideration

Sample size calculation was based on the method developed by Frison and Pocock using STATA SE 10 (STATA, College Station, TX) [22]. Parameters used for sample size calculation were on the basis of our pilot trial and documented data from the metabolic patients in our clinics. We recruited 190 subjects (95 per group) to have 80% power at 5% significance level; to account for a potential attrition rate of 20%, we finalized a total of 240 subjects.

### Statistical analysis

Chi-squared test were performed for categorical variables. Continuous variables were presented as means ± standard error (S.E.). Sodium intake and sodium density were evaluated using linear mixed models, which were used to examine the differences between baseline and one-year visits, meal location, meal type, and meal time (weekdays versus weekends). Four models were fitted in the analyses of meal location and day of week. In Model 1, independent variables included time-points, meal type, location, gender, study condition, the day of week, and the interaction of time-points, meal type and the day of week. In Model 2 when sodium intake was fitted as dependent variable, meal location was removed to test the difference between weekdays and weekend days. Model 3 and Model 4 adjusted the caloric intake based on Model 1 and Model 2, respectively. Differences were considered significant at *p* <0.05. Statistical analyses were performed using SAS 9.2 software (SAS Institute, Cary, North Carolina).

## Results

**Figure 1:**
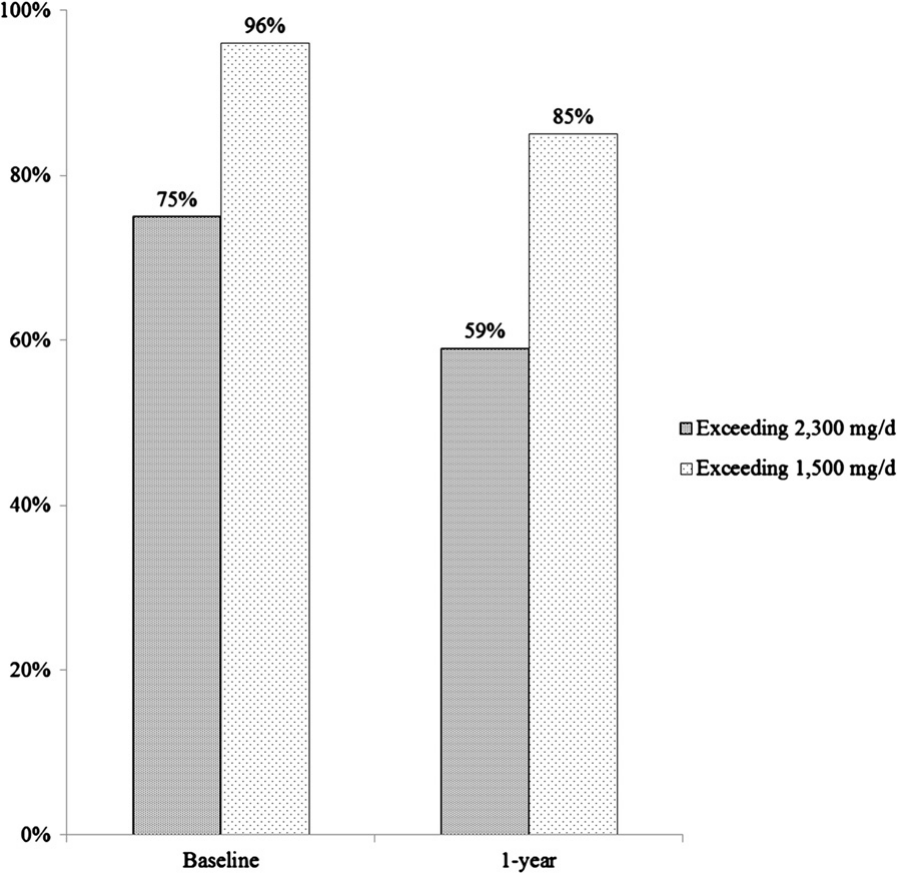
The percentage of excess sodium intake over time points.

Two hundred and forty obese subjects participated in the present study [mean and standard deviation (SD): BMI: 35 ± 3 kg/m^2^] and met at least three components of MetS. The average age was 52 (SD = 10) years old and 72% were women. Figure 1 shows the improvement of sodium intake at baseline and one-year visits among participants at the 1500 and 2300 mg/d limits recommended by the DGA and the AHA, respectively [8,20,23]. The percentage of excess sodium intake decreased (*p* < 0.001) at each cut-point.

Sodium intake stratified by selected demographic characteristics is presented in Table 1. The average sodium intake decreased from 2994 ± 72 mg/d at baseline to 2558 ± 77 mg/d at one-year visit (*p* < 0.001); the sodium potassium ratio also decreased from 1.211 ±0.027 to 1.047 ± 0.031. At baseline, sodium consumption was significantly higher among subjects who were younger than 51 y, male, and employed (*p* < 0.001; *p* = 0.014; *p* = 0.004, respectively). At the one-year visit, sodium intake was consistently reduced; however a significant difference was only observed between males (3051 ± 146 mg/d) and females (2380 ± 88 mg/d; p < 0.001 Table 1 also shows that there were no statistically significant differences between the AHA and high fiber diet groups for daily sodium intake or sodium density at both baseline and at one year.

**Table 1:** **Sodium intake by participants’ characteristics**^**1**^

**Variable**	**Baseline**		**One year**		***P *** **for time-points**^**2**^
	**n**	**Mean (S.E.) (mg/day)**	**n**	**Mean (S.E.) (mg/day)**	
**Sodium intake (mg/day)**	240	2994 (72)	184	2558 (77)	<0.001
**Sodium density (mg/kcal)**	240	1.613 (0.025)	184	1.660 (0.034)	0.249
**Sodium/potassium ratio**	240	1.211 (0.027)	184	1.047 (0.031)	<0.001
**Treatment arms**					
** AHA diet**	119	3063 (99)	92	2415 (110)	<0.001
** High fiber diet**	121	2927 (98)	92	2694 (110)	
*** p value***^**3**^		0.330		0.074	
**Gender**					
** Female**	173	2742 (77)	135	2380 (88)	<0.001
** Male**	67	3645 (125)	49	3051 (146)	
*** p value***^**3**^		<0.001		<0.001	
**Age group**					
** <51 y**	93	3211 (112)	59	2546 (140)	<0.001
** ≥51 y**	147	2857 (89)	125	2564 (96)	
*** p value***^**3**^		0.014		0.916	
**Race/ethnicity**					
** Caucasian**	213	3034 (74)	165	2545 (84)	0.145
** Others**	27	2683 (208)	19	2670 (248)	
*** p value***^**3**^		0.115		0.634	
**Education**					
** High school diploma or less**	33	2989 (189)	25	2843 (217)	0.005
** Bachelor’s degree or less**	146	3054 (90)	108	2561 (104)	
** Graduate/professional**	59	2833 (141)	49	2398 (155)	
*** p value***^**3**^		0.509		0.095	
**Household income**					
** $0-$30000**	24	3104 (220)	15	2617 (270)	<0.001
** $30000-$50000**	43	3167 (165)	36	2567 (180)	
** $50000-$75000**	40	2851 (171)	33	2345 (188)	
** More than $75000**	79	3167 (122)	60	2612 (139)	
** Unclear**	54	2662 (147)	40	2625 (171)	
*** p value***^**3**^		0.096		0.419	
**Currently working**					
** Full-time or part-time**	188	3101(78)	145	2550 (89)	0.027
** Others**	52	2607 (149)	39	2590 (172)	
*** p value***^**3**^		0.004		0.837	
**Marital status**					
** Married**	162	2979 (85)	121	2590 (99)	<0.001
** Not Married**	78	3026 (123)	63	2498 (137)	
*** p value***^**3**^		0.753		0.584	
**Components of metabolic syndrome**					
*** 3***	71	2820 (126)	52	2573 (166)	<0.001
*** 4***	96	3171 (108)	76	2639 (126)	
*** 5***	72	2902 (125)	44	2446 (166)	
*** p value***^**3**^		0.096		0.796	
**Dietary attitudes to low sodium**					
**Agree limiting sodium is important**	195	2914 (76)	169	2531 (82)	0.008
**Not sure/disagree sodium is important**	44	3322 (155)	15	2928 (258)	
*** P value***^***3***^		0.017		0.141	

^1^Sodium intakes per meal described with mean ± standard error and were estimated by LSMEANS of PROC MIXED model in SAS.

^2^*P* values for time-points were compared at baseline and 1-year and determined from mixed models fitting item, time-points and interaction between them. However, none of the interaction terms were significant in the models.

^3^*P* values compared the differences between groups at the same time-point and were determined from LSMEANS of PROC MIXED model in SAS.

In addition, dietary attitudes might also play a role in decisions regarding sodium consumption. At baseline, the 44 participants (18.4%) who were ambivalent or disagreed that limiting sodium is important had a higher sodium intake (3322 ± 155 mg/d) compared to those who ranked sodium intake as important (2914 ± 76 mg/d; p = 0.017). One-year after the intervention, sodium intake was consistently reduced regardless of whether subjects agreed with this concept.

To increase statistical power, we used pooled data for further analyses. Sodium consumption (mg) and sodium density (mg/kcal) by meal type, location, and weekday are presented in Table 2. In general, the majority of participants ate most of their meals at home with the majority of their sodium intake coming from dinner at home and when eating at restaurant/fast food chains anytime. Their sodium intake declined (except when eating at restaurant/fast food chains) after the one-year intervention.

**Table 2:** **Sodium intake (mg/meal) and sodium density (mg/kcal) by meal type, weekday and location at baseline and one year **^**1**^

	**Breakfast**		**Lunch**		**Dinner**		**Snack**	
**Location**^**2,7**^	**Baseline**	**One-year**	**Baseline**	**One-year**	**Baseline**	**One-year**	**Baseline**	**One-year**
**At home**								
	*n = 273*	*n = 275*	*n = 184*	*n = 171*	*n = 409*	*n = 330*	*n = 695*	*n = 535*
Sodium intake	487 ± 39	425 ± 39	1062 ± 47^4^	883 ± 49	1281 ± 33^4^	1058 ± 36	228 ± 26	215 ± 30
Sodium density	1.29 ± 0.05	1.28 ± 0.06	2.22 ± 0.11	2.36 ± 0.12	1.94 ± 0.06	1.91 ± 0.07	1.02 ± 0.04	1.29 ± 0.14
**Away from home**								
	*n = 67*	*n = 59*	*n = 140*	*n = 131*	*n = 19*	*n = 18*	*n = 302*	*n = 220*
Sodium intake	527 ± 77	394 ± 82	1102 ± 54^4^	841 ± 56	1317 ± 142	1132 ± 146	169 ± 38	193 ± 45
Sodium density	1.38 ± 0.09	1.22 ± 0.13	2.13 ± 0.09	2.04 ± 0.12	2.30 ± 0.24	2.26 ± 0.34	0.94 ± 0.07	0.95 ± 0.08
**Restaurant/fast food**								
	*n = 25*	*n = 15*	*n = 65*	*n = 57*	*n = 62*	*n = 59*	*n = 37*	*n = 9*
Sodium intake	1023 ± 124^5^	881 ± 161^5^	1478 ± 78^5^	1488 ± 83^5^	1610 ± 80^4,5^	1861 ± 82^5^	274 ± 103	670 ± 206^5^
Sodium density	1.68 ± 0.12^5^	1.85 ± 0.19^5^	2.19 ± 0.13	2.29 ± 0.13	1.80 ± 0.13	2.00 ± 0.14	0.81 ± 0.15	1.36 ± 0.40
**Weekdays**^**3,7**^								
	*n = 225*	*n = 248*	*n = 249*	*n = 254*	*n = 307*	*n = 279*	*n = 680*	*n = 554*
Sodium intake	488 ± 44	413 ± 42	1098 ± 42^4^	948 ± 41	1256 ± 38^4^	1137 ± 40	168 ± 27	200 ± 30
Sodium density	1.32 ± 0.05	1.28 ± 0.06	2.22 ± 0.09	2.26 ± 0.09	1.91 ± 0.07	1.93 ± 0.08	0.93 ± 0.04	1.25 ± 0.13
**Weekend days**^**3,7**^								
	*n = 140*	*n = 101*	*n = 140*	*n = 105*	*n = 183*	*n = 128*	*n = 354*	*n = 210*
Sodium intake	587 ± 54	464 ± 64	1213 ± 54^4^	965 ± 63	1420 ± 48^4,6^	1233 ± 57	271 ± 36^6^	204 ± 46
Sodium density	1.37 ± 0.07	1.33 ± 0.10	2.11 ± 0.10	2.16 ± 0.13	1.97 ± 0.10	1.96 ± 0.11	1.09 ± 0.07	1.05 ± 0.10

^1^Sodium intakes per meal and sodium density per meal described with mean ± standard error and were estimated by LSMEANS of PROC MIXED model in SAS.

^2^Model 1: Tested by Proc Mixed model in SAS, fitting sodium intake or sodium density as the dependent variable, independent variables included meal type (*P* < 0.001), day of week (*P* < 0.001), time-point (*P* = 0.238), location (*P* < 0.001), gender (*P* < 0.001), condition (*P* = 0.836) and interaction of meal and location and time-point (*P* < 0.001) as fixed effects, and subject ID as a random effect.

^3^Model 2: Tested by Proc Mixed model in SAS, fitting sodium intake or sodium density as dependent variable, independent variables included meal type (*P* < 0.001), day of week (*P* < 0.001), time-point (*P* < 0.001), gender (*P* < 0.001), condition (*P* = 0.802) and interaction of meal and weekday and time-point (*P* = 0.024) as fixed effects, and subject ID as a random effect.

^4^*P* < 0.05 and P values compared differences between baseline and one year and were determined from LSMEANS of PROC MIXED model in SAS.

^5^*P* < 0.05 and P values compared differences between restaurant/fast food and eaten at home at the same time-point and determined from LSMEANS of PROC MIXED model in SAS.

^6^*P* < 0.05 and P values compared differences to weekdays and determined from LSMEANS of PROC MIXED in SAS.

^7^For sodium intake, Models 3 and 4 were additionally adjusted for total caloric intake and all aforementioned statistically significant differences were attenuated towards the null.

After the one-year intervention, subjects consumed lower amounts of sodium when they ate at home with significant differences observed at lunch (1062 ± 47 versus 883 ± 49 mg; p < 0.05) and dinner (1281 ± 33 versus 1058 ± 36 mg; p < 0.05). Sodium intake when subjects ate away from home also decreased after the one-year intervention; however, a significant difference was only observed at lunch (1102 ± 54 versus 841 ± 56 mg; p < 0.05). Compared to baseline, sodium intake after the one-year intervention increased when subjects ate at restaurants/fast food chains with a significant difference for dinner (1610 ± 80 versus 1861 ± 82 mg; p < 0.05). However, the sodium density per meal between meal locations and the day of week were similar except for breakfast when consumed away from home or at restaurant/fast food (Baseline:1.68 ± 0.12 versus 1.29 ± 0.05 mg/kcal; p < 0.05; One-year: 1.85 ± 0.19 versus 1.28 ± 0.06 mg/kcal; p < 0.05).

## Discussion

Previous studies have reported that over 83% of Americans consume sodium higher than 2300 mg per day [8,24,25]. Our analysis identified dietary locations and meals that may be targeted to improve the excessive sodium intake among people with metabolic syndrome. We observed that sodium intake significantly declined during our study intervention similar to the DASH diet findings (2558 mg/d versus 2473 mg/d) [26]; however, the decrease may be a result of reduced total energy intake since sodium density did not favorably change. Moreover, the sodium density remained similar for every location of meals consumed, even when meals eaten at home might have been be easier to manipulate.

Our one-year dietary intervention helped metabolic syndrome participants reduce sodium consumption; however their intakes remained much higher than the 1500 mg. AHA recommendation. Much like the findings from the National Health and Nutrition Examination Survey (NHANES) [9,27], our participants chose to consume processed foods including bread and rolls, cold cuts/cured meats, pizza, poultry, soups, sandwiches, cheese, etc. (data not shown). Further, similar to other studies, our study found that mean sodium consumption was significantly greater for foods obtained from fast food or other restaurants and stores, indicating that sodium consumption mostly comes not only from table salt, but particularly salt added to processed foods [8,9,25,28]. Since raw/fresh foods contain much less sodium than prepared or processed foods [29], the Dietary Guidelines for Americans (2010) suggest that people eat more fresh and home-prepared foods, eat fewer processed foods, and select lower sodium items at restaurants. However, to better understand and follow these recommendations requires a very knowledgeable and motivated consumer able to identify and choose low sodium foods at restaurants. Since many foods consumed already have salt added, instructing individuals to cut back on added salt is unlikely to achieve the dietary guidelines.

Informed individual choices and population-based interventions are important approaches to reduce sodium consumption [30]. Simply recommending a reduction in sodium without support from the overall food suppliers is unlikely to achieve the desired outcomes without a significant decrease in calories, which may not be appropriate for every adult. Rather, a change in dietary sodium intake requires strong public health policies directed at the reduction of sodium in commercial processing, accompanied by lifestyle initiatives that encourage the preparation of higher quality foods using fresh ingredients without added sodium.

Policies for food and menu labeling have been proposed to improve the food environment. However, overall sodium consumption remains high in the U.S. [31]. To achieve meaningful sodium reductions and to help consumers make healthful choices, we need additional strategies that increase the availability of lower sodium products and reduce the amount of sodium in foods served outside the home, while expanding educational efforts that facilitate these healthy choices [32,33].

The attention to salt reduction has been increased worldwide, with eleven countries in the European Union on board to make a 16% reduction in salt intake over the next 4 years [34]. Several countries, including the United Kingdom (UK) and Finland, with epidemiologic surveillance and front-of-package ”traffic-light” sodium labeling, have successfully carried out salt reduction programs [35,36]. The U.S. may consider these and other strategies in order to impact high sodium intake and its sequelae of health consequences [37].

Our study has several limitations. First, our participants were obese adults (BMI between 30 and 40) with MetS, our sample is also limited, and therefore our findings may not be generalizable to the overall U.S. population. Second, this study measured sodium intake via 24-hour dietary recalls. Self-reported dietary intake may be affected by recall bias and sodium intake is more likely to be underestimated [4,38]. Third, the AHA has emphasized that the goal of sodium intake is 1500 mg/d or less, but few people in our study met this threshold. This study also calculated compliance to the less stringent upper level of 2300 mg/d, which was the prior 2006 AHA recommendation [8,20,23,39]. Still, few of our participants achieved this goal. While research generally agrees that the lower level of sodium is beneficial to circulatory diseases, the practical reduction of sodium intake may require a step-wise approach to reduce excess sodium intake a realistic and achievable goal for the U.S. population. Finally, since our study was not focused exclusively on reducing sodium, this may contribute to the fact that there was no change in sodium density.

The present study also has several strengths. To our knowledge, this is the first study that reported detailed sodium intake and meal consumption patterns in a dietary intervention trial. In addition, three 24-hour dietary recalls were conducted to collect dietary data at baseline and one-year, which may be more precise than food frequency questionnaires [40].

## Conclusions

This study observed a high percentage of participants who consumed excess sodium even after a dietary intervention directed in part to increase awareness of intake among obese subjects with MetS. Not surprisingly, the sodium intake was higher on weekend days, at lunch and dinner, and when meals were consumed in restaurants and fast food chains. Although the percentage of excess sodium intake decreased after the one-year intervention, sodium reduction was likely accomplished through a decrease in total caloric intake. If the aim of public policy is to reduce sodium intake independently, decreasing the sodium density of commercially prepared food in addition to limiting added salt is necessary. Findings from this study highlight the role of public health policies in helping individuals meet the guidelines and to encourage people to prepare low sodium foods at home.

## Competing interests

The authors declare that they have no competing interests.

## Authors’ contributions

JW, BCO, and YM designed the research; BCO, PAM, GFO, and YM carried out the research; JW and YM wrote the draft of initial manuscript; JW, BCO, NMW, GMP, ALC, WL, PAM, JC, HF, ZZ, GFO, LZ and YM critically revised the manuscript for important intellectual content; JW, GFO, and HF analyzed data; PAM and JC provided the essential administrative, technical, or material support. YM and BCO had primary responsibility for final content. All authors read and approved the final manuscript.
